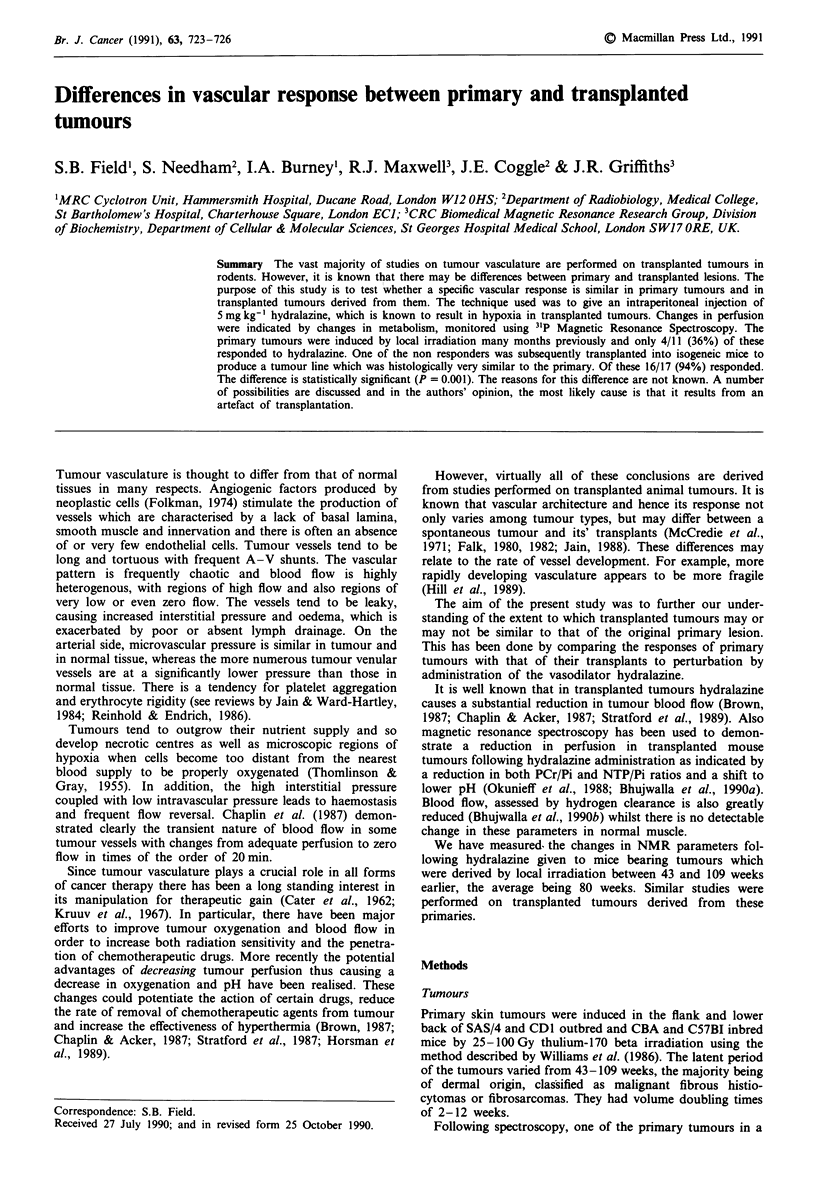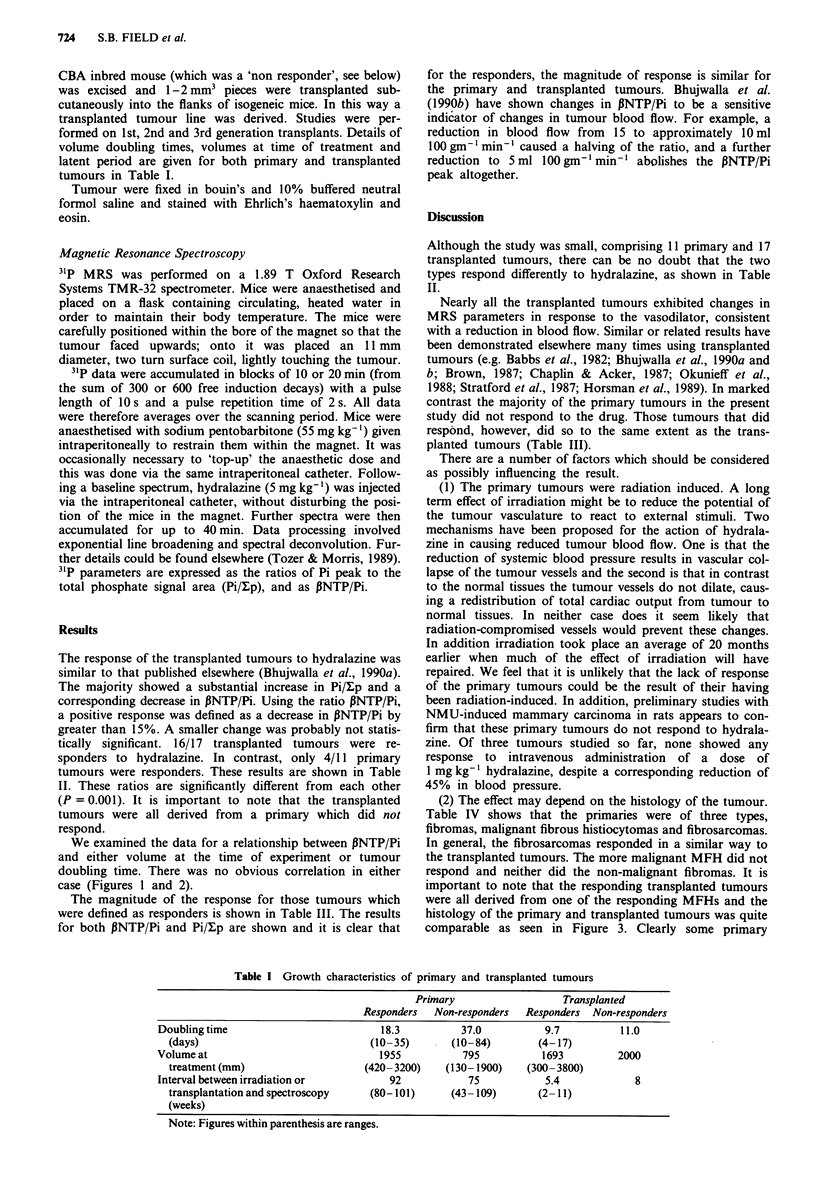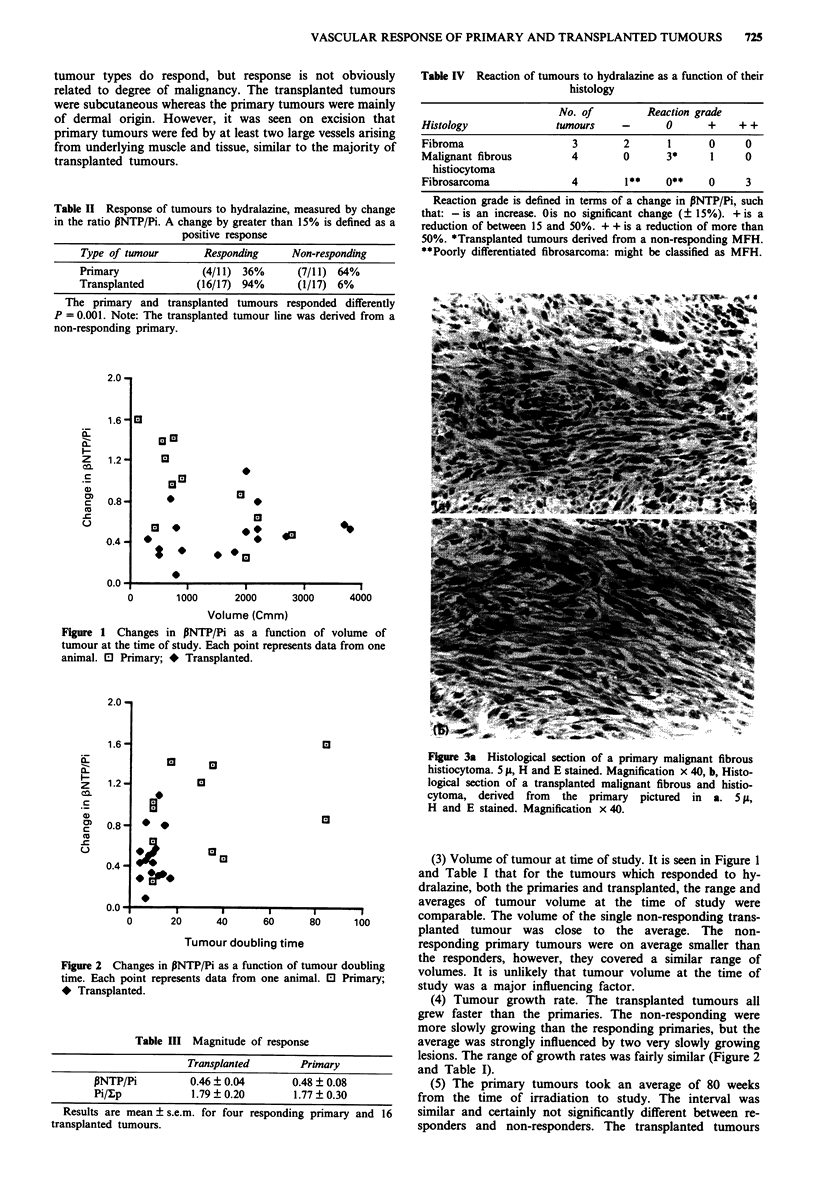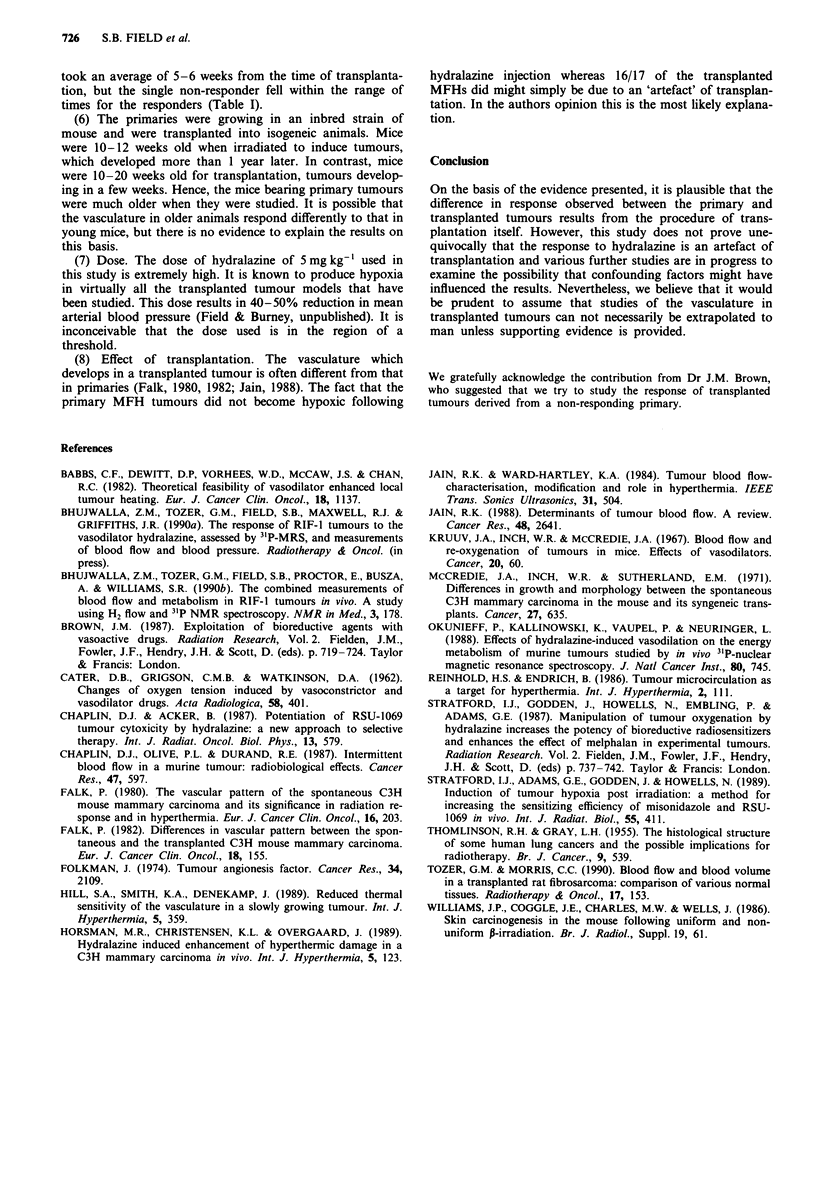# Differences in vascular response between primary and transplanted tumours.

**DOI:** 10.1038/bjc.1991.163

**Published:** 1991-05

**Authors:** S. B. Field, S. Needham, I. A. Burney, R. J. Maxwell, J. E. Coggle, J. R. Griffiths

**Affiliations:** MRC Cyclotron Unit, Hammersmith Hospital, London, UK.

## Abstract

**Images:**


 © Macmillan Press Ltd., 1991

### Differences in vascular response between primary and transplanted tumours

S.B. Field[1], S. Needham[2], I.A. Burney[1], R.J. Maxwell[3], J.E. Coggle[2] & J.R. Griffiths[3]

[1]*MRC Cyclotron Unit, Hammersmith Hospital, Ducane Road, London W12 0HS;* [2]*Department of Radiobiology, Medical College, St Bartholomew's Hospital, Charterhouse Square, London EC1;* [3]*CRC Biomedical Magnetic Resonance Research Group, Division of Biochemistry, Department of Cellular & Molecular Sciences, St Georges Hospital Medical School, London SW17 0RE, UK.*

**Summary** The vast majority of studies on tumour vasculature are performed on transplanted tumours in rodents. However, it is known that there may be differences between primary and transplanted lesions. The purpose of this study is to test whether a specific vascular response is similar in primary tumours and in transplanted tumours derived from them. The technique used was to give an intraperitoneal injection of $5\ mg\ kg^{-1}$ hydralazine, which is known to result in hypoxia in transplanted tumours. Changes in perfusion were indicated by changes in metabolism, monitored using $^{31}P$ Magnetic Resonance Spectroscopy. The primary tumours were induced by local irradiation many months previously and only 4/11 (36%) of these responded to hydralazine. One of the non responders was subsequently transplanted into isogeneic mice to produce a tumour line which was histologically very similar to the primary. Of these 16/17 (94%) responded. The difference is statistically significant ($P = 0.001$). The reasons for this difference are not known. A number of possibilities are discussed and in the authors' opinion, the most likely cause is that it results from an artefact of transplantation.

Tumour vasculature is thought to differ from that of normal tissues in many respects. Angiogenic factors produced by neoplastic cells (Folkman, 1974) stimulate the production of vessels which are characterised by a lack of basal lamina, smooth muscle and innervation and there is often an absence of or very few endothelial cells. Tumour vessels tend to be long and tortuous with frequent A–V shunts. The vascular pattern is frequently chaotic and blood flow is highly heterogenous, with regions of high flow and also regions of very low or even zero flow. The vessels tend to be leaky, causing increased interstitial pressure and oedema, which is exacerbated by poor or absent lymph drainage. On the arterial side, microvascular pressure is similar in tumour and in normal tissue, whereas the more numerous tumour venular vessels are at a significantly lower pressure than those in normal tissue. There is a tendency for platelet aggregation and erythrocyte rigidity (see reviews by Jain & Ward-Hartley, 1984; Reinhold & Endrich, 1986).

Tumours tend to outgrow their nutrient supply and so develop necrotic centres as well as microscopic regions of hypoxia when cells become too distant from the nearest blood supply to be properly oxygenated (Thomlinson & Gray, 1955). In addition, the high interstitial pressure coupled with low intravascular pressure leads to haemostasis and frequent flow reversal. Chaplin *et al.* (1987) demonstrated clearly the transient nature of blood flow in some tumour vessels with changes from adequate perfusion to zero flow in times of the order of 20 min.

Since tumour vasculature plays a crucial role in all forms of cancer therapy there has been a long standing interest in its manipulation for therapeutic gain (Cater *et al.*, 1962; Kruuv *et al.*, 1967). In particular, there have been major efforts to improve tumour oxygenation and blood flow in order to increase both radiation sensitivity and the penetration of chemotherapeutic drugs. More recently the potential advantages of *decreasing* tumour perfusion thus causing a decrease in oxygenation and pH have been realised. These changes could potentiate the action of certain drugs, reduce the rate of removal of chemotherapeutic agents from tumour and increase the effectiveness of hyperthermia (Brown, 1987; Chaplin & Acker, 1987; Stratford *et al.*, 1987; Horsman *et al.*, 1989).

However, virtually all of these conclusions are derived from studies performed on transplanted animal tumours. It is known that vascular architecture and hence its response not only varies among tumour types, but may differ between a spontaneous tumour and its' transplants (McCredie *et al.*, 1971; Falk, 1980, 1982; Jain, 1988). These differences may relate to the rate of vessel development. For example, more rapidly developing vasculature appears to be more fragile (Hill *et al.*, 1989).

The aim of the present study was to further our understanding of the extent to which transplanted tumours may or may not be similar to that of the original primary lesion. This has been done by comparing the responses of primary tumours with that of their transplants to perturbation by administration of the vasodilator hydralazine.

It is well known that in transplanted tumours hydralazine causes a substantial reduction in tumour blood flow (Brown, 1987; Chaplin & Acker, 1987; Stratford *et al.*, 1989). Also magnetic resonance spectroscopy has been used to demonstrate a reduction in perfusion in transplanted mouse tumours following hydralazine administration as indicated by a reduction in both PCr/Pi and NTP/Pi ratios and a shift to lower pH (Okunieff *et al.*, 1988; Bhujwalla *et al.*, 1990*a*). Blood flow, assessed by hydrogen clearance is also greatly reduced (Bhujwalla *et al.*, 1990*b*) whilst there is no detectable change in these parameters in normal muscle.

We have measured the changes in NMR parameters following hydralazine given to mice bearing tumours which were derived by local irradiation between 43 and 109 weeks earlier, the average being 80 weeks. Similar studies were performed on transplanted tumours derived from these primaries.

### Methods

### *Tumours*

Primary skin tumours were induced in the flank and lower back of SAS/4 and CD1 outbred and CBA and C57Bl inbred mice by 25–100 Gy thulium-170 beta irradiation using the method described by Williams *et al.* (1986). The latent period of the tumours varied from 43–109 weeks, the majority being of dermal origin, classified as malignant fibrous histiocytomas or fibrosarcomas. They had volume doubling times of 2–12 weeks.

Following spectroscopy, one of the primary tumours in a

Correspondence: S.B. Field.
Received 27 July 1990; and in revised form 25 October 1990.

CBA inbred mouse (which was a 'non responder', see below) was excised and 1–2 $mm^3$ pieces were transplanted subcutaneously into the flanks of isogeneic mice. In this way a transplanted tumour line was derived. Studies were performed on 1st, 2nd and 3rd generation transplants. Details of volume doubling times, volumes at time of treatment and latent period are given for both primary and transplanted tumours in Table I.

Tumour were fixed in bouin's and 10% buffered neutral formol saline and stained with Ehrlich's haematoxylin and eosin.

*Magnetic Resonance Spectroscopy*

$^{31}P$ MRS was performed on a 1.89 T Oxford Research Systems TMR-32 spectrometer. Mice were anaesthetised and placed on a flask containing circulating, heated water in order to maintain their body temperature. The mice were carefully positioned within the bore of the magnet so that the tumour faced upwards; onto it was placed an 11 mm diameter, two turn surface coil, lightly touching the tumour.

$^{31}P$ data were accumulated in blocks of 10 or 20 min (from the sum of 300 or 600 free induction decays) with a pulse length of 10 s and a pulse repetition time of 2 s. All data were therefore averages over the scanning period. Mice were anaesthetised with sodium pentobarbitone (55 mg $kg^{-1}$) given intraperitoneally to restrain them within the magnet. It was occasionally necessary to 'top-up' the anaesthetic dose and this was done via the same intraperitoneal catheter. Following a baseline spectrum, hydralazine (5 mg $kg^{-1}$) was injected via the intraperitoneal catheter, without disturbing the position of the mice in the magnet. Further spectra were then accumulated for up to 40 min. Data processing involved exponential line broadening and spectral deconvolution. Further details could be found elsewhere (Tozer & Morris, 1989). $^{31}P$ parameters are expressed as the ratios of Pi peak to the total phosphate signal area (Pi/Σp), and as βNTP/Pi.

### Results

The response of the transplanted tumours to hydralazine was similar to that published elsewhere (Bhujwalla *et al.*, 1990*a*). The majority showed a substantial increase in Pi/Σp and a corresponding decrease in βNTP/Pi. Using the ratio βNTP/Pi, a positive response was defined as a decrease in βNTP/Pi by greater than 15%. A smaller change was probably not statistically significant. 16/17 transplanted tumours were responders to hydralazine. In contrast, only 4/11 primary tumours were responders. These results are shown in Table II. These ratios are significantly different from each other ($P = 0.001$). It is important to note that the transplanted tumours were all derived from a primary which did *not* respond.

We examined the data for a relationship between βNTP/Pi and either volume at the time of experiment or tumour doubling time. There was no obvious correlation in either case (Figures 1 and 2).

The magnitude of the response for those tumours which were defined as responders is shown in Table III. The results for both βNTP/Pi and Pi/Σp are shown and it is clear that for the responders, the magnitude of response is similar for the primary and transplanted tumours. Bhujwalla *et al.* (1990*b*) have shown changes in βNTP/Pi to be a sensitive indicator of changes in tumour blood flow. For example, a reduction in blood flow from 15 to approximately 10 ml 100 $gm^{-1}$ $min^{-1}$ caused a halving of the ratio, and a further reduction to 5 ml 100 $gm^{-1}$ $min^{-1}$ abolishes the βNTP/Pi peak altogether.

### Discussion

Although the study was small, comprising 11 primary and 17 transplanted tumours, there can be no doubt that the two types respond differently to hydralazine, as shown in Table II.

Nearly all the transplanted tumours exhibited changes in MRS parameters in response to the vasodilator, consistent with a reduction in blood flow. Similar or related results have been demonstrated elsewhere many times using transplanted tumours (e.g. Babbs *et al.*, 1982; Bhujwalla *et al.*, 1990*a* and *b*; Brown, 1987; Chaplin & Acker, 1987; Okunieff *et al.*, 1988; Stratford *et al.*, 1987; Horsman *et al.*, 1989). In marked contrast the majority of the primary tumours in the present study did not respond to the drug. Those tumours that did respond, however, did so to the same extent as the transplanted tumours (Table III).

There are a number of factors which should be considered as possibly influencing the result.

(1) The primary tumours were radiation induced. A long term effect of irradiation might be to reduce the potential of the tumour vasculature to react to external stimuli. Two mechanisms have been proposed for the action of hydralazine in causing reduced tumour blood flow. One is that the reduction of systemic blood pressure results in vascular collapse of the tumour vessels and the second is that in contrast to the normal tissues the tumour vessels do not dilate, causing a redistribution of total cardiac output from tumour to normal tissues. In neither case does it seem likely that radiation-compromised vessels would prevent these changes. In addition irradiation took place an average of 20 months earlier when much of the effect of irradiation will have repaired. We feel that it is unlikely that the lack of response of the primary tumours could be the result of their having been radiation-induced. In addition, preliminary studies with NMU-induced mammary carcinoma in rats appears to confirm that these primary tumours do not respond to hydralazine. Of three tumours studied so far, none showed any response to intravenous administration of a dose of 1 mg $kg^{-1}$ hydralazine, despite a corresponding reduction of 45% in blood pressure.

(2) The effect may depend on the histology of the tumour. Table IV shows that the primaries were of three types, fibromas, malignant fibrous histiocytomas and fibrosarcomas. In general, the fibrosarcomas responded in a similar way to the transplanted tumours. The more malignant MFH did not respond and neither did the non-malignant fibromas. It is important to note that the responding transplanted tumours were all derived from one of the responding MFHs and the histology of the primary and transplanted tumours was quite comparable as seen in Figure 3. Clearly some primary

**Table I** Growth characteristics of primary and transplanted tumours

| | *Primary* | | *Transplanted* | |
|---|---|---|---|---|
| | *Responders* | *Non-responders* | *Responders* | *Non-responders* |
| Doubling time | 18.3 | 37.0 | 9.7 | 11.0 |
| (days) | (10–35) | (10–84) | (4–17) | |
| Volume at | 1955 | 795 | 1693 | 2000 |
| treatment (mm) | (420–3200) | (130–1900) | (300–3800) | |
| Interval between irradiation or | 92 | 75 | 5.4 | 8 |
| transplantation and spectroscopy | (80–101) | (43–109) | (2–11) | |
| (weeks) | | | | |

Note: Figures within parenthesis are ranges.

tumour types do respond, but response is not obviously related to degree of malignancy. The transplanted tumours were subcutaneous whereas the primary tumours were mainly of dermal origin. However, it was seen on excision that primary tumours were fed by at least two large vessels arising from underlying muscle and tissue, similar to the majority of transplanted tumours.

**Table II** Response of tumours to hydralazine, measured by change in the ratio βNTP/Pi. A change by greater than 15% is defined as a positive response

| *Type of tumour* | *Responding* | *Non-responding* |
|---|---|---|
| Primary | (4/11) 36% | (7/11) 64% |
| Transplanted | (16/17) 94% | (1/17) 6% |

The primary and transplanted tumours responded differently $P = 0.001$. Note: The transplanted tumour line was derived from a non-responding primary.

**Figure 1** Changes in βNTP/Pi as a function of volume of tumour at the time of study. Each point represents data from one animal. ☐ Primary; ◆ Transplanted.

**Figure 2** Changes in βNTP/Pi as a function of tumour doubling time. Each point represents data from one animal. ☐ Primary; ◆ Transplanted.

**Table III** Magnitude of response

| | *Transplanted* | *Primary* |
|---|---|---|
| βNTP/Pi | 0.46 ± 0.04 | 0.48 ± 0.08 |
| Pi/Σp | 1.79 ± 0.20 | 1.77 ± 0.30 |

Results are mean ± s.e.m. for four responding primary and 16 transplanted tumours.

**Table IV** Reaction of tumours to hydralazine as a function of their histology

| *Histology* | *No. of tumours* | *Reaction grade* – | 0 | + | + + |
|---|---|---|---|---|---|
| Fibroma | 3 | 2 | 1 | 0 | 0 |
| Malignant fibrous histiocytoma | 4 | 0 | 3* | 1 | 0 |
| Fibrosarcoma | 4 | 1** | 0** | 0 | 3 |

Reaction grade is defined in terms of a change in βNTP/Pi, such that: – is an increase. 0 is no significant change (± 15%). + is a reduction of between 15 and 50%. + + is a reduction of more than 50%. *Transplanted tumours derived from a non-responding MFH. **Poorly differentiated fibrosarcoma: might be classified as MFH.

**Figure 3a** Histological section of a primary malignant fibrous histiocytoma. 5 μ, H and E stained. Magnification × 40, **b**, Histological section of a transplanted malignant fibrous and histiocytoma, derived from the primary pictured in **a**. 5 μ, H and E stained. Magnification × 40.

(3) Volume of tumour at time of study. It is seen in Figure 1 and Table I that for the tumours which responded to hydralazine, both the primaries and transplanted, the range and averages of tumour volume at the time of study were comparable. The volume of the single non-responding transplanted tumour was close to the average. The non-responding primary tumours were on average smaller than the responders, however, they covered a similar range of volumes. It is unlikely that tumour volume at the time of study was a major influencing factor.

(4) Tumour growth rate. The transplanted tumours all grew faster than the primaries. The non-responding were more slowly growing than the responding primaries, but the average was strongly influenced by two very slowly growing lesions. The range of growth rates was fairly similar (Figure 2 and Table I).

(5) The primary tumours took an average of 80 weeks from the time of irradiation to study. The interval was similar and certainly not significantly different between responders and non-responders. The transplanted tumours took an average of 5–6 weeks from the time of transplantation, but the single non-responder fell within the range of times for the responders (Table I).

(6) The primaries were growing in an inbred strain of mouse and were transplanted into isogeneic animals. Mice were 10–12 weeks old when irradiated to induce tumours, which developed more than 1 year later. In contrast, mice were 10–20 weeks old for transplantation, tumours developing in a few weeks. Hence, the mice bearing primary tumours were much older when they were studied. It is possible that the vasculature in older animals respond differently to that in young mice, but there is no evidence to explain the results on this basis.

(7) Dose. The dose of hydralazine of $5 \text{ mg kg}^{-1}$ used in this study is extremely high. It is known to produce hypoxia in virtually all the transplanted tumour models that have been studied. This dose results in 40–50% reduction in mean arterial blood pressure (Field & Burney, unpublished). It is inconceivable that the dose used is in the region of a threshold.

(8) Effect of transplantation. The vasculature which develops in a transplanted tumour is often different from that in primaries (Falk, 1980, 1982; Jain, 1988). The fact that the primary MFH tumours did not become hypoxic following hydralazine injection whereas 16/17 of the transplanted MFHs did might simply be due to an 'artefact' of transplantation. In the authors opinion this is the most likely explanation.

### Conclusion

On the basis of the evidence presented, it is plausible that the difference in response observed between the primary and transplanted tumours results from the procedure of transplantation itself. However, this study does not prove unequivocally that the response to hydralazine is an artefact of transplantation and various further studies are in progress to examine the possibility that confounding factors might have influenced the results. Nevertheless, we believe that it would be prudent to assume that studies of the vasculature in transplanted tumours can not necessarily be extrapolated to man unless supporting evidence is provided.

We gratefully acknowledge the contribution from Dr J.M. Brown, who suggested that we try to study the response of transplanted tumours derived from a non-responding primary.

### References

BABBS, C.F., DEWITT, D.P, VORHEES, W.D., MCCAW, J.S. & CHAN, R.C. (1982). Theoretical feasibility of vasodilator enhanced local tumour heating. *Eur. J. Cancer Clin. Oncol.*, **18**, 1137.

BHUJWALLA, Z.M., TOZER, G.M., FIELD, S.B., MAXWELL, R.J. & GRIFFITHS, J.R. (1990*a*). The response of RIF-1 tumours to the vasodilator hydralazine, assessed by $^{31}$P-MRS, and measurements of blood flow and blood pressure. *Radiotherapy & Oncol.* (in press).

BHUJWALLA, Z.M., TOZER, G.M., FIELD, S.B., PROCTOR, E., BUSZA, A. & WILLIAMS, S.R. (1990*b*). The combined measurements of blood flow and metabolism in RIF-1 tumours *in vivo*. A study using $H_2$ flow and $^{31}$P NMR spectroscopy. *NMR in Med.*, **3**, 178.

BROWN, J.M. (1987). Exploitation of bioreductive agents with vasoactive drugs. *Radiation Research*, Vol. 2. Fielden, J.M., Fowler, J.F., Hendry, J.H. & Scott, D. (eds). p. 719–724. Taylor & Francis: London.

CATER, D.B., GRIGSON, C.M.B. & WATKINSON, D.A. (1962). Changes of oxygen tension induced by vasoconstrictor and vasodilator drugs. *Acta Radiologica*, **58**, 401.

CHAPLIN, D.J. & ACKER, B. (1987). Potentiation of RSU-1069 tumour cytoxicity by hydralazine: a new approach to selective therapy. *Int. J. Radiat. Oncol. Biol. Phys.*, **13**, 579.

CHAPLIN, D.J., OLIVE, P.L. & DURAND, R.E. (1987). Intermittent blood flow in a murine tumour: radiobiological effects. *Cancer Res.*, **47**, 597.

FALK, P. (1980). The vascular pattern of the spontaneous C3H mouse mammary carcinoma and its significance in radiation response and in hyperthermia. *Eur. J. Cancer Clin. Oncol.*, **16**, 203.

FALK, P. (1982). Differences in vascular pattern between the spontaneous and the transplanted C3H mouse mammary carcinoma. *Eur. J. Cancer Clin. Oncol.*, **18**, 155.

FOLKMAN, J. (1974). Tumour angionesis factor. *Cancer Res.*, **34**, 2109.

HILL, S.A., SMITH, K.A., DENEKAMP, J. (1989). Reduced thermal sensitivity of the vasculature in a slowly growing tumour. *Int. J. Hyperthermia*, **5**, 359.

HORSMAN, M.R., CHRISTENSEN, K.L. & OVERGAARD, J. (1989). Hydralazine induced enhancement of hyperthermic damage in a C3H mammary carcinoma *in vivo*. *Int. J. Hyperthermia*, **5**, 123.

JAIN, R.K. & WARD-HARTLEY, K.A. (1984). Tumour blood flow-characterisation, modification and role in hyperthermia. *IEEE Trans. Sonics Ultrasonics*, **31**, 504.

JAIN, R.K. (1988). Determinants of tumour blood flow. A review. *Cancer Res.*, **48**, 2641.

KRUUV, J.A., INCH, W.R. & MCCREDIE, J.A. (1967). Blood flow and re-oxygenation of tumours in mice. Effects of vasodilators. *Cancer*, **20**, 60.

MCCREDIE, J.A., INCH, W.R. & SUTHERLAND, E.M. (1971). Differences in growth and morphology between the spontaneous C3H mammary carcinoma in the mouse and its syngeneic transplants. *Cancer*, **27**, 635.

OKUNIEFF, P., KALLINOWSKI, K., VAUPEL, P. & NEURINGER, L. (1988). Effects of hydralazine-induced vasodilation on the energy metabolism of murine tumours studied by *in vivo* $^{31}$P-nuclear magnetic resonance spectroscopy. *J. Natl Cancer Inst.*, **80**, 745.

REINHOLD, H.S. & ENDRICH, B. (1986). Tumour microcirculation as a target for hyperthermia. *Int. J. Hyperthermia*, **2**, 111.

STRATFORD, I.J., GODDEN, J., HOWELLS, N., EMBLING, P. & ADAMS, G.E. (1987). Manipulation of tumour oxygenation by hydralazine increases the potency of bioreductive radiosensitizers and enhances the effect of melphalan in experimental tumours. *Radiation Research*. Vol. 2. Fielden, J.M., Fowler, J.F., Hendry, J.H. & Scott, D. (eds) p. 737–742. Taylor & Francis: London.

STRATFORD, I.J., ADAMS, G.E., GODDEN, J. & HOWELLS, N. (1989). Induction of tumour hypoxia post irradiation: a method for increasing the sensitizing efficiency of misonidazole and RSU-1069 *in vivo*. *Int. J. Radiat. Biol.*, **55**, 411.

THOMLINSON, R.H. & GRAY, L.H. (1955). The histological structure of some human lung cancers and the possible implications for radiotherapy. *Br. J. Cancer.*, **9**, 539.

TOZER, G.M. & MORRIS, C.C. (1990). Blood flow and blood volume in a transplanted rat fibrosarcoma: comparison of various normal tissues. *Radiotherapy & Oncol.*, **17**, 153.

WILLIAMS, J.P., COGGLE, J.E., CHARLES, M.W. & WELLS, J. (1986). Skin carcinogenesis in the mouse following uniform and non-uniform β-irradiation. *Br. J. Radiol.*, Suppl. 19, 61.